# Phylogeography reveals an ancient cryptic radiation in East-Asian tree frogs (*Hyla japonica* group) and complex relationships between continental and island lineages

**DOI:** 10.1186/s12862-016-0814-x

**Published:** 2016-11-23

**Authors:** Christophe Dufresnes, Spartak N. Litvinchuk, Amaël Borzée, Yikweon Jang, Jia-Tang Li, Ikuo Miura, Nicolas Perrin, Matthias Stöck

**Affiliations:** 1 Department of Ecology and Evolution, Biophore Building, University of Lausanne, 1015 Lausanne, Switzerland; 2 Institute of Cytology, Russian Academy of Sciences, Tikhoretsky pr. 4, 194064 St. Petersburg, Russia; 3 Laboratory of Behavioral Ecology and Evolution, School of Biological Sciences, Seoul National University, 151-747 Seoul, Republic of Korea; 4 Division of EcoScience, Ewha Womans University, Seoul, 120-750 Republic of Korea; 5 Chengdu Institute of Biology, Chinese Academy of Sciences, Chengdu, 610041 China; 6 Institute for Amphibian Biology, Graduate School of Science, Hiroshima University, Kagamiyama 1-3-1, Higashihiroshima, 739-8526 Japan; 7 Leibniz-Institute of Freshwater Ecology and Inland Fisheries (IGB), Müggelseedamm 301, D-12587 Berlin, Germany; 8 Visiting Research Fellow at the Graduate School of Science, Hiroshima University, Hiroshima, Japan

**Keywords:** Amphibian conservation, Eastern Palearctics, Hylidae, Phylogeography, Refugia within refugia, Quaternary glaciations

## Abstract

**Background:**

In contrast to the Western Palearctic and Nearctic biogeographic regions, the phylogeography of Eastern-Palearctic terrestrial vertebrates has received relatively little attention. In East Asia, tectonic events, along with Pleistocene climatic conditions, likely affected species distribution and diversity, especially through their impact on sea levels and the consequent opening and closing of land-bridges between Eurasia and the Japanese Archipelago. To better understand these effects, we sequenced mitochondrial and nuclear markers to determine phylogeographic patterns in East-Asian tree frogs, with a particular focus on the widespread *H. japonica*.

**Results:**

We document several cryptic lineages within the currently recognized *H. japonica* populations, including two main clades of Late Miocene divergence (~5 Mya). One occurs on the northeastern Japanese Archipelago (Honshu and Hokkaido) and the Russian Far-East islands (Kunashir and Sakhalin), and the second one inhabits the remaining range, comprising southwestern Japan, the Korean Peninsula, Transiberian China, Russia and Mongolia. Each clade further features strong allopatric Plio-Pleistocene subdivisions (~2-3 Mya), especially among continental and southwestern Japanese tree frog populations. Combined with paleo-climate-based distribution models, the molecular data allowed the identification of Pleistocene glacial refugia and continental routes of postglacial recolonization. Phylogenetic reconstructions further supported genetic homogeneity between the Korean *H. suweonensis* and Chinese *H. immaculata*, suggesting the former to be a relic population of the latter that arose when the Yellow Sea formed, at the end of the last glaciation.

**Conclusions:**

Patterns of divergence and diversity were likely triggered by Miocene tectonic activities and Quaternary climatic fluctuations (including glaciations), causing the formation and disappearance of land-bridges between the Japanese islands and the continent. Overall, this resulted in a ring-like diversification of *H. japonica* around the Sea of Japan. Our findings urge for important taxonomic revisions in East-Asian tree frogs. First, they support the synonymy of *H. suweonensis* (Kuramoto, 1980) and *H. immaculata* (Boettger, 1888). Second, the nominal *H. japonica* (Günther, 1859) represents at least two species: an eastern (new taxon A) on the northern Japanese and Russian Far East islands, and a southwestern species (n. t. B) on southern Japanese islands and possibly also forming continental populations. Third, these continental tree frogs may also represent an additional entity, previously described as *H. stepheni* Boulenger, 1888 (senior synonym of *H. ussuriensis* Nikolskii, 1918). A complete revision of this group requires further taxonomic and nomenclatural analyses, especially since it remains unclear to which taxon the species-epitheton *japonica* corresponds to.

**Electronic supplementary material:**

The online version of this article (doi:10.1186/s12862-016-0814-x) contains supplementary material, which is available to authorized users.

## Background

Historical biogeography is now fairly well-understood in the Western Palearctic realm, where the location of glacial refugia, routes of postglacial recolonization and the geoclimatic processes that shaped the current genetic diversity have been inferred in a wide array of terrestrial vertebrates (reviewed in: [1–4]). In contrast, the Eastern Palearctic, and notably the East-Asian region, also called ‘Sino-Japanese’, has received less attention both in term of the number of investigated species and the geological timescales covered [1, 2]. This area encompasses Eastern China, East Siberia and Far-Eastern Russia, Mongolia, the Korean Peninsula and the Japanese Archipelago. During the Tertiary, the terrestrial biodiversity of this region was influenced by faunal exchanges via the major crossroad between Eurasia and North-America: the Beringian land-bridge [2, 5]. Within the East-Asian region, phylogeographers further identified old diversifications between the Chinese mainland, the Korean Peninsula and the Japanese Archipelago (e.g. [6–8]), as well as younger differentiation within these regions (e.g. [9–12]), consistent with major geological events of the Miocene and Pliocene (e.g. subduction of the Philippine Sea tectonic plate, expansion of ancient basins). The impact of more recent processes, namely the Quaternary glaciations, is also poorly understood. Compared to Europe and North America, Pleistocene climatic conditions were supposedly milder in NE-Asia, but still affected species distributions and diversity [8, 13, 14]. Especially, the Pleistocene climatic fluctuations had strong impact on sea levels, involving coastline changes and the opening and closing of land-bridges between Eurasia and the Japanese Archipelago [15, 16]. For instance, Komaki et al*.* [17] recently compared mitochondrial and nuclear phylogenies in context to paleoclimatic modeling in East Asian water frogs (*Pelophylax*) from Far-Eastern Russia, China, the Japan, Korea and Taiwan and detected five historical mtDNA introgressions and range shifts, all considered to be shaped by glacial cycling.

Phylogeography can inform about the natural history and evolutionary processes that shaped specific and intraspecific diversity in this biogeographically important region. Moreover, knowledge about the distribution of current diversity has direct conservation applications, both for the delineation of management units [18] and to appreciate the potential vulnerability of genetically impoverished populations [19].

Generally, potentially slow-dispersing ectotherms like amphibians are known to show strong genetic responses to historical climatic changes and geological processes (e.g. [20, 21]). Here we focus on the East-Asian tree frogs (*Hyla japonica* group; recently changed to *Dryophytes japonicus * [22]), which offer an excellent opportunity to advance our historic biogeographic knowledge on this region. The *H. japonica* group has resulted from the second wave of hylid colonization of Eurasia that invaded during Early to Middle Miocene (95% 21–15 Mya) from the Nearctic [5]. It currently comprises at least three recognized species [23]: the Japanese tree frog *H. japonica* (Günther, 1859 [24]), which has a wide continental (Eastern China, Eastern Russia and Mongolia, Korean Peninsula) and insular (Japanese Archipelago, Kurily and Sakhalin Islands) distribution; the spotless tree frog *H. immaculata* (Boettger, 1888 [25]), endemic to Eastern China; and the endangered Suweon tree frog *H. suweonensis* (Kuramoto, 1980 [26]), narrowly distributed and endemic to northwestern South Korea. Furthermore, some authors have considered *H. japonica*’s mainland populations as a separate species (*H. stepheni*, Boulenger 1888 (“1887”), a senior synonym of *H. ussuriensis*, Nikolsky, 1918 [27]), which, however, appears unsupported by recent molecular data [5]. In a recent phylogeny of Holarctic *Hyla*, these authors [5] hypothesized potential discrepancies in the current systematics of these taxa: based on few mitochondrial sequences, they suspected cryptic insular speciation on the Russian eastern islands of Sakhalin and Kunashir (inhabited by *H. japonica*), as well possibly synonymy between *H. immaculata* and *H. suweonensis*. Therefore, more detailed investigations are required to shed light on cryptic diversity and clarify the evolutionary relationships in this species group [5], as well as to understand the ecological and evolutionary processes involved.

In this paper, we aim to understand the diversification patterns of East Asian tree frogs and whether divergences correspond to major biogeographic events in the region. Specifically, we undertake a phylogeographic survey of *H. japonica* across its continental and insular range, and explore phylogenetic relationships with its relatives *H. immaculata* and *H. suweonensi*s. To do so, we sequenced fast-evolving mitochondrial and nuclear markers from samples across most of the range of *H. japonica* and estimate divergence times between all major lineages. To complement the phylogeographic analyses and get insights into the location of glacial refugia, we estimated the current and past distribution for the widespread *H. japonica* using Species Distribution Modelling approaches. Given that tree frogs are generally declining and threatened in many regions, our results also have potentially useful implications for the future management of populations.

## Methods

### DNA sampling, extraction and marker sequencing

Tissue or oral ("buccal") swab samples were obtained from museum samples (ZISP, Zoological Institute of the Russian Academy of Sciences, St. Petersburg; CIB, Chengdu Institute of Biology, the Chinese Academy of Sciences) and field captured frogs. For the latter, DNA was obtained from muscle or liver, or from non-lethal sampling (toe clips, oral swabs), in which case the animals were immediately released afterwards. A total of 96 specimens of *H. japonica* from 34 populations were considered (28 from ZISP, nine from CIB, 57 newly collected, two from GenBank). Two samples from *H. immaculata* (newly collected) and seven from *H. suweonensis* (three newly collected, four from GenBank) were included. Details on the origin of specimens are available from Additional file 1. All specimens were collected according to local permits and approved by ethics committees in the various countries. DNA was extracted using the Qiagen DNeasy Blood and Tissue extraction kit.

We sequenced ~900 bp of the mitochondrial *cytochrome-b* (*cyt-b*), in all individuals as described [28]. For nuclear data, we amplified and cloned a ~500 bp part *α-Fibrinogen* intron 1 (methods and cross-amplifying primers: [29]) in a representative subset of individuals (*H. japonica*: *n* = 36, *H. immaculata*: *n* = 2; we included published sequences for *H. suweonensis*, see below). At least eight clones were sequenced per sample in order to capture the two alleles of heterozygotes.

### Phylogenetic analyses

We calculated maximum likelihood phylogenies of *cyt-b* haplotypes with PhyML [30], using a GTR + G + I model of sequence evolution (MrAIC, [31]). For this analysis, we also included published *cyt-b* sequences of *H. suweonensis* (*n* = 10 haplotypes isolated from 68 individuals from the entire narrow range of this species, [32]; GenBank KF564855-KF564864), as well as a sequence of *H. chinensis* as outgroup (GenBank NC006403). We performed a similar analysis for the nuclear *α-Fibrinogen* haplotypes, using a HKY + I model (MrAIC) and included our published sequences for *H. suweonensis* (*n* = 1 haplotype after alignment, out of 4 individuals cloned and sequenced, [29]; GenBank KR816385-KR816392). A sequence of *H. annectans* was used as outgroup (GenBank JX182365; [28]). For both phylogenetic trees, branch support was tested by 1000 bootstrap replicates.

We estimated divergence times between the main East-Asian tree frog lineages with BEAST [33], using the molecular calibration of the Western Palearctic radiation of *Hyla* by Stöck et al*.* [28]. For this purpose, we included representative *cyt-b* sequences, available for seven European taxa (3–11 per taxon) with a resolved phylogeny and known divergence times (*H. meridionalis*, *H. savignyi*, *H. felixarabica*, *H. arborea*, *H. intermedia*, *H. molleri* and *H. orientalis*). Importantly, the *cyt-b* tree is concordant with the most robust phylogenetic analyses for these species published to date, based on thousands of SNPs [29]. We applied calibration points to the split of *H. meridionalis* (9.7 Mya), of the *H. arborea* group (6.1 Mya) and between *H. molleri* and *H. orientalis* (3.7 Mya), using normally distributed priors with a standard deviation of 1 My. This enables to effectively span a large range of prior distributions to encompass the posterior distributions obtained by Stöck et al*.* [28] for these estimates. We applied a Yule tree prior, as appropriate for species-level divergences, and ran two chains, each comprising 30 million iterations. We then used Tracer [34] to verify for high ESS values, and checked for convergence and stationary of the results. The first 3 million runs were discarded as ‘Burnin’. A maximum clade credibility tree was built using the BEAST module TreeAnnotator, available as Additional file 2. In addition, we computed genetic distances (*Dxy*) between and within the main mtDNA clades with DNAsp [35]. Given its low polymorphism and unresolved topology (see Results), we did not perform molecular dating of *α-Fibrinogen* haplogroups.

Finally, to explore the genetic structure of *H. japonica* populations, we built networks of mitochondrial and nuclear haplotypes using TCS [36], without restriction on the maximal numbers of connections. For *α-Fibrinogen*, we considered insertion-deletion (indel) polymorphism and the two alleles of each sample separately.

### Past distribution modelling of *H. japonica*

For the contemporary niche predictions, we used 1008 localities of *H. japonica* (Additional file 3), combining our sampling localities (Additional file 1), the Global Biodiversity Information Facility database [37], and published monitoring data [38, 39]. Duplicated localities were removed by ENMTools 1.3 [40]. For the present time and the Last Glacial Maximum (LGM; about 22,000 years ago), 19 bioclimatic layers (2.5 min resolution) were extracted from the WorldClim database [41]. Two general atmospheric circulation models were used to generate LGM climate scenarios: the Community Climate System Model (CCSM, [42]) and the Model for Interdisciplinary Research on Climate (MIROC; [43]). Models were generated by Maxent 3.3.3 [44, 45], using default settings. We used 70% of the occurrence localities as training data, and the remaining 30% were reserved for testing the resulting models. We evaluated our predicted models using Area Under the Curve (AUC) derived from the Receiver Operating Characteristic plots. The plots represent a model’s ability to discriminate species locations from pseudo-absences by plotting sensitivity against 1 - specificity [46]. AUC values range from 0.5 to 1.0, with 0.5 indicating no greater fit than expected by chance and 1.0 indicating a perfect model fit. Models with test AUC values above 0.75 are considered useful and above 0.90 very good [47, 48]. For the prediction of potential geographic distributions, correlations between pairs of bioclimatic variables were assessed using the Pearson correlation coefficient by ENMTools, in order to avoid highly correlated and redundant variables. Two variables sharing a correlation coefficient of 0.8 or higher were considered highly correlated. Previous knowledge on biology and requirements of the studied species is crucial for optimal modeling [49]. We therefore selected variables based on known preferences of *Hyla* species. After correcting for correlation among data layers, seven variables were retained: Bio1 (annual mean temperature; °C × 10), Bio2 (mean diurnal range; °C × 10), Bio3 (isothermality), Bio5 (maximum temperature of warmest month; °C × 10), Bio12 (annual precipitation; mm), Bio15 (precipitation seasonality; CV), and Bio19 (precipitation of coldest quarter; mm). We used a jackknife analysis for estimation of relative contributions of the variables to the Maxent model. The mask applied for the distribution modelling extends from 90 to 155° N and 20 to 65° E.

## Results

Mitochondrial and nuclear trees recovered similar haplogroups, although topologies differed in respect to the lower informativeness of the nuclear marker, which accordingly also featured low branch supports. The mitochondrial *cyt-b* was very informative: we sampled 67 haplotypes (containing 220 variable sites, 202 of which parsimony-informative). For the nuclear *α-Fibrinogen*, we distinguished 28 haplotypes with 42 variable sites, including 22 parsimony-informative.

### Relationships of East-Asian tree frogs

**Fig. 1:**
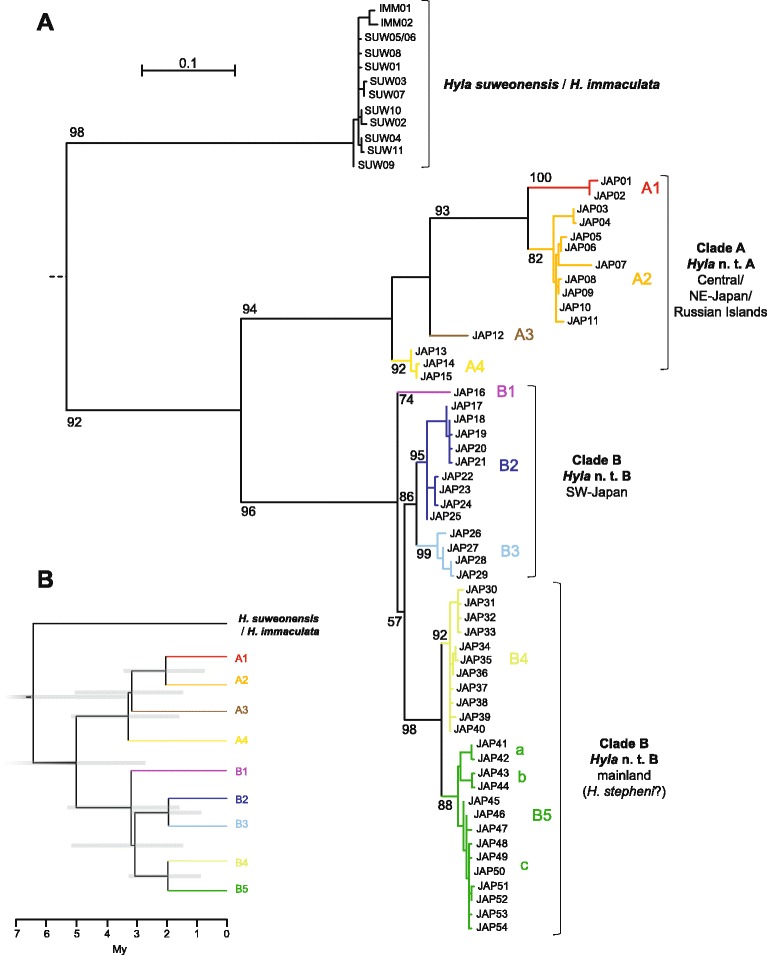
**a** Maximum-likelihood mitochondrial phylogeny of Eastern Asian tree frogs, based on *cyt-b* haplotypes; **b** Chronogram of divergences. Bootstrap support is indicated for major nodes, if above 50%. 95% HPD of divergence times are given for major nodes on the chronogram. For the widespread continental subclade B5, we distinguished the three haplogroups that have strong geographic association and probably testify of different glacial subrefugia. N. t.: new taxon

**Fig. 2:**
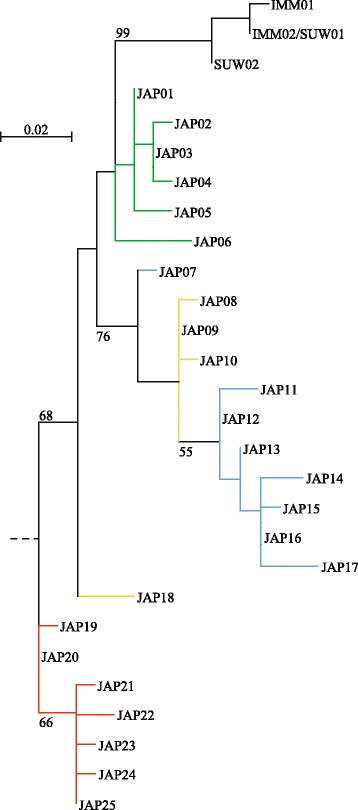
Maximum-likelihood phylogeny of the nuclear *α-Fibrinogen* haplotypes. Colors correspond to the major mtDNA lineages. Bootstrap support is indicated for major nodes, if above 50%

In both phylogenetic trees, *H. immaculata* sequences unambiguously clustered with those of *H. suweonensis*, forming a genetically homogeneous monophyletic clade (Figs. 1 and 2). In the mitochondrial tree (Fig. 1), this clade forms a sister clade to the *H. japonica* radiation, with the time to their most recent common ancestor estimated at ~6.4 Mya (95% HPD: 10.3-3.3 My; *Dxy* = 0.13, Additional file 4). In the nuclear tree, the *H. immaculata*/*H. suweonensis* haplogroup is highly supported (99% bootstrap support, Fig. 2), although it appears paraphyletic in respect to *H. japonica*.

### Diversification of Japanese tree frogs (*Hyla japonica*)

**Fig. 3:**
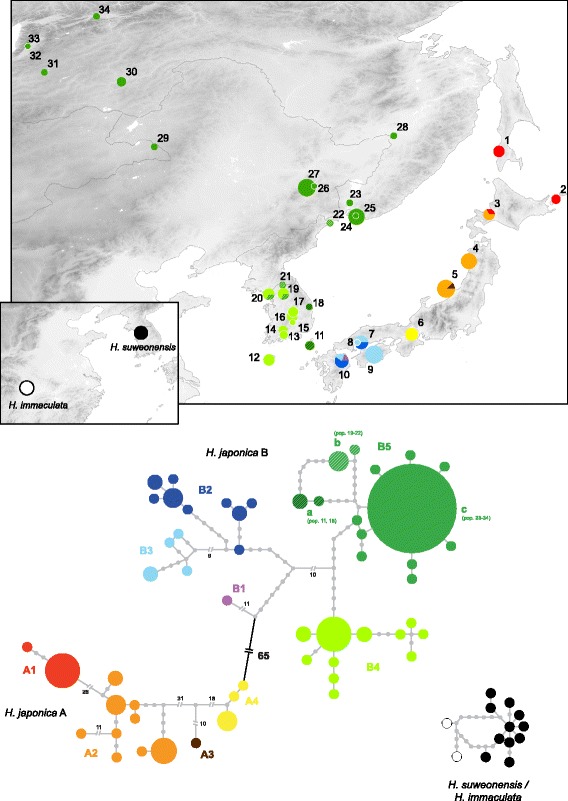
Haplotype network of mitochondrial *cyt-b* and distribution of the main lineages. For *H. japonica*, pies are proportional to sample sizes and colors correspond to the different clades. Haplotypes of *H. suweonensis* (ours and GenBank data) were all sampled within the vicinity of Seoul, Korea, where it is endemic

The mitochondrial phylogenetic tree recovered a very diverse, old but monophyletic radiation of *H. japonica*, including at least nine mitochondrial lineages with varying degrees of divergence (Fig. 1) and clear geographic association (Fig. 3).

Specifically, we distinguished two major clades, diverged ~5.0 Mya (95% HPD: 7.9-2.7 My; *Dxy* = 0.104, Additional file 4):

(i). The first clade (A) is restricted to the central and northern parts of the Japanese Archipelago, Kunashir and Sakhalin Islands (pop. 1–6), and is further subdivided into four main lineages (*Dxy* from 0.036 to 0.070, Additional file 4) found on Sakhalin, Kunashir and Hokkaido (subclade A1, red; pop. 1–3), northern Honshu and southern Hokkaido Islands (subclade A2, orange; pop. 3–5), Sado Island (subclade A3, brown; pop. 5) and stretching to central Honshu (subclade A4, yellow; pop. 6).
(ii). the second clade (B) has a wide distribution across the southern parts of the Japanese Archipelago and the mainland (pop. 7–34), with marked subdivisions (*Dxy* from 0.017 to 0.042, Additional file 4) in southern Japan (subclades B1, purple; B2, dark blue; and B3, light blue; pop. 7–10), southwest of the Korean Peninsula (subclade B4, light green; pop. 12–20) and the remaining part of its continental range (subclade B5, dark green; pop. 18–34). Divergence time estimates suggest two additional periods for these diversifications: some 3 Mya (splits of subclades A4, A3 and B1; estimated at ~3.3-3.2 Mya), and some 2 Mya (splits of subclades A1, A2, B2, B3, B4 and B5; estimated at ~2.1-2.0 Mya).

Figure 1 provides all estimates and their HPD. The mitochondrial haplotype network (Fig. 3) confirms the strong signals of population clustering and further highlights haplotype diversity for some subclades (i.e. A2, B2, B3) and star-like patterns for haplogroups of the continental subclades B4 and B5 (Fig. 3). In clade B5, this involves haplotypes segregating in the northern parts of the range (B5c, pop. 23–34), whereas distinct haplotypes occur in Korea (B5a, B5b; Fig. 3).

**Fig. 4:**
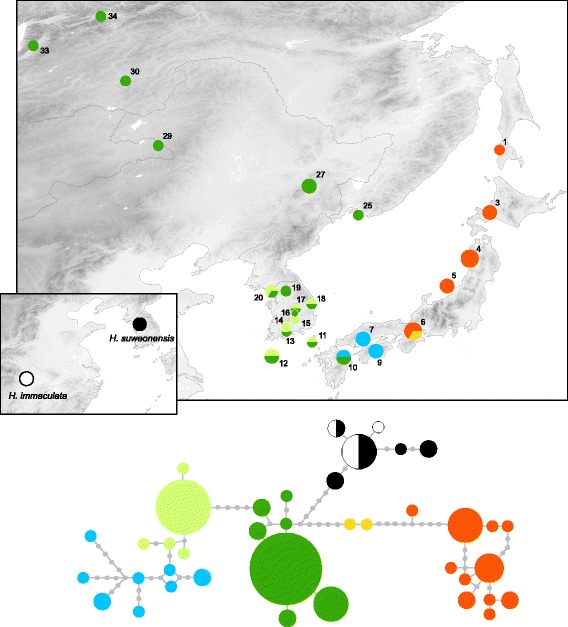
Haplotype network of nuclear *α-Fibrinogen* and distribution of nuclear haplogroups. Pies are proportional to sample sizes. For *H. japonica*, colors correspond to the equivalent mitochondrial clades/subclades. Haplotypes of *H. immaculata*/*H. suweonensis* are colored according to which species there were sampled from (*H. immaculata*: white, *H. suweonensis*: black: both: black/white)

Nuclear phylogenetic analyses and haplotype network recovered most of *H. japonica* mitochondrial genetic groups, however with some unresolved relationships (Figs. 2 and 4). Samples, belonging to mitochondrial clade A, form the basal branches of the nuclear tree (nuclear haplotypes JAP18-25) with subclade A4 standing out (yellow, JAP18). Frogs of mitochondrial clade B are represented by paraphyletic haplogroups that distinguish mtDNA-subclades B4 (nuclear haplotypes JAP08-10) and B5 (nuclear haplotypes JAP01-06) from southern Japanese ones (mitochondrial subclades B1-3; nuclear haplotypes JAP07, JAP11-17). As for mtDNA, the nuclear haplotype network forms star-like patterns for continental haplogroups, whereas the Japanese samples present a more complex structure.

### Historic distribution modelling for *H. japonica*

**Fig. 5:**
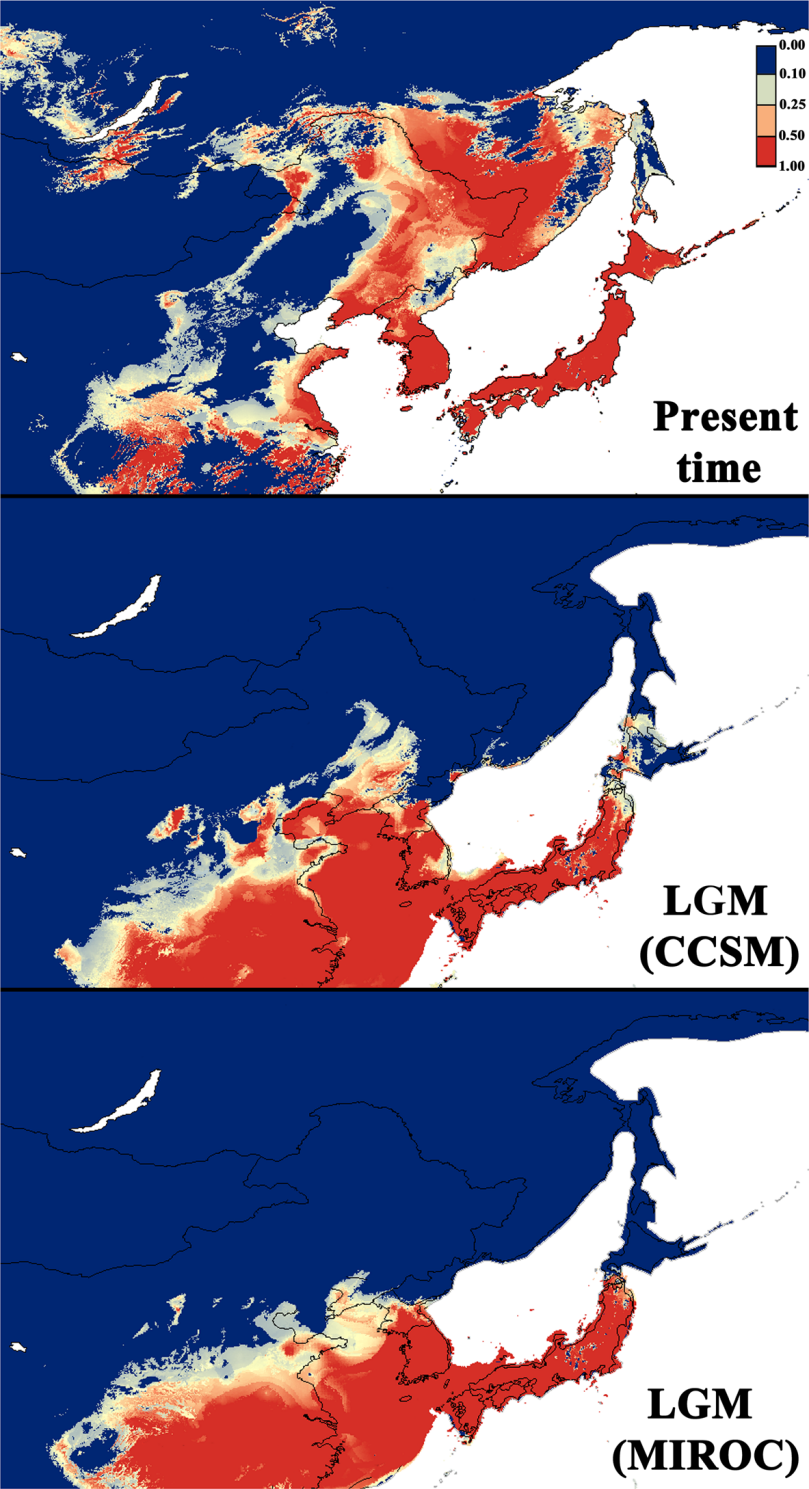
Present and past distribution models for the Last Glacial Maximum (CCSM, MIROC) in *H. japonica*

The ecological niche modeling had high AUC test values (0.935 ± 0.005) and showed significance for the binomial omission test, indicating a good performance of the model. The predicted potential niche model under the current climate conditions is shown in Fig. 5. Four variables (Bio12, Bio1, Bio3, and Bio5) accounted for 90% of the predicted range. The projected potential niche models for the LGM indicate a substantial southward retraction of the range for *H. japonica*. The extent of range shrinkage is variable depending on the Global Circulation Model. According to the CCSM model, the suitable environmental conditions for the species were widely distributed across the Japanese Archipelago, Tsushima, Sado, and Jeju Islands, the southernmost part of Sakhalin Island, most of the Korea Peninsula, the shallow Yellow Sea, the southern Manchuria and the southernmost part of Primorskiy kray of Russia. According to the MIROC model, the suitable conditions were somewhat less widely distributed: namely, across the Japanese Archipelago (excluding Hokkaido), Tsushima, Sado, and Jeju islands, most of the Korea Peninsula, the shallow Yellow Sea, and the southernmost Manchuria.

## Discussion

**Fig. 6:**
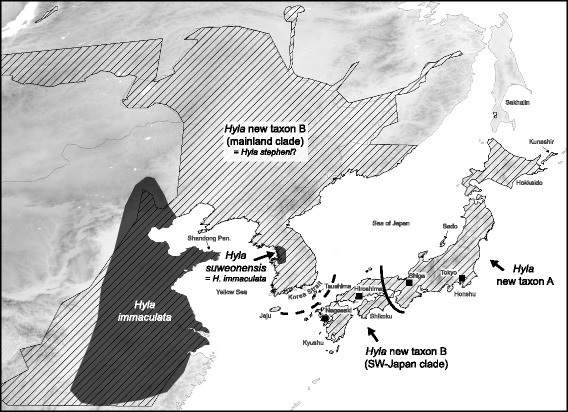
Distribution ranges of Eastern Asian tree frogs with proposed splits of taxa based on our molecular analyses. We split the currently recognized “*Hyla japonica*” as *Hyla* new taxa A and B (cf. Fig. 1), in dashed areas. The distribution of *H. immaculata* and its Korean synonym *H. suweonensis* are displayed in plain dark. Localities mentioned in this study are indicated. Distribution data: IUCN red list

Mitochondrial and nuclear phylogenetic analyses of tree frogs from the East-Asian mainland and the Japanese Archipelago revealed three main lineages, including cryptic genetic diversity within *H. japonica*. The latter represents a species group, composed of two major genetic clades (corresponding to two new taxa A and B) with pronounced subdivisions. Figure 6 illustrates their distribution ranges and proposes taxonomic revisions based on our molecular data. In the following, we discuss biogeographic and systematic implications.

### Diversification and historical biogeography of East-Asian tree frogs

Molecular dating based on the mitochondrial *cyt-b* suggested a Late Miocene divergence (~6.5 Mya) of the common ancestor of *H. immaculata* + *H. suweonensis* from the lineage leading to all current nominal *H. japonica*. This branching corresponds to the geological period when the Philippine Sea Plate (PHS) resumed its subduction, forming large volcano-tectonic depressions across the Korean Strait (separating the Japanese Archipelago from the Korean Peninsula) and the Ryuku Arc (separating Japan from mainland China; [50]), eventually breaking land-bridges that formerly connected the Japanese Archipelago with the continent [51]. These geological events may have played a role in isolating other ancient Japanese and continental vertebrate species, as other amphibians [9], fishes [12], and especially mammals, which consequently feature high levels of endemism in Japan [16]. In accordance with mtDNA, nuclear sequences supported a distinct *H. immaculata*/*H. suweonensis* haplogroup, although nuclear phylogenetic relationships with *H. japonica* could not be resolved based solely on this marker, likely due to its low polymorphism.

Our findings show that the widespread *H. japonica* harbors high cryptic diversity throughout its range with multiple Miocene and Plio-Pleistocene diversifications. This genetically diverse taxon can first be divided into two main mitochondrial clades (Fig. 1), with the corresponding nuclear differentiation, as well as disjoint distributions, one occurring on the eastern Japanese and Far-Eastern Russian islands (mtDNA-clade A, n. t. A), the other distributed across southern Japan and the remaining range (mtDNA-clade B, n. t. B). Previous analyses, based on allozyme polymorphisms, accordingly recovered strong genetic differentiation between southern and northern Japanese *H. japonica* populations [52]. The old divergence between these two mtDNA-clades (~5 Mya) may be linked to past geological processes of the Early Pliocene, namely the expansion of ancient basins in central Japan, with associated volcanic activity, as evidenced in the Setouchi geological province [53]. This same event seems to have caused similar divergences between southwestern and northeastern Japan in other anurans, such as *Rana rugosa* [11], *Buergeria buergeri* [54] and *Bufo* toads [9]. From our data, we predict the two *H. japonica* clades A and B to come into secondary contact in the region spanning from Shiga and Hiroshima Prefectures (between pop. 6 and 7). Further population genetic investigations will probably inform on the accurate distribution of these taxa and on their genetic interactions across contact zones.

Each *H. japonica* mtDNA-clade further presents allopatric subdivisions, of Plio-Pleistocene divergence (2–3 Mya). In clade A (n. t. A), the complex topography of central of northern Japan, combined with Pleistocene climatic fluctuations, may have promoted this strong geographical structure. Especially, subclade A1, occurring on Hokkaido, Kunashir and Sakhalin Islands may have become isolated during unsuitable Quaternary glacial periods, as suggested by the CCSM past distribution model (Fig. 5). In mtDNA-clade B (n. t. B), southern Japanese tree frogs are also highly genetically diverse, both on the mitochondrial and nuclear levels. In Japan, the co-occurrence of diverged haplotypes within single populations (e.g. pop. 3, 5, 7, 10) may testify of a complex dynamic of allopatric differentiations (for instance, when sea level disconnected islands during interglacial phases, or when mountain ranges isolated population during glacial periods), alternating with population reconnection and gene flow. Therefore, the diversification throughout Japan likely reflects the existence of multiple Pleistocene refugia, paralleling the situation of other Japanese amphibians [9, 11, 17], mammals [10] and fishes [12]. In mammals, Pleistocene climatic fluctuations had a strong impact on population extinction and inter-island dispersal and recolonization [16].

Molecular dating within mtDNA-clade B (n. t. B) shows that continental populations diverged from southern Japanese ones about 3 Mya. The mitochondrial differentiation was also accompanied by the formation of distinct nuclear haplogroups. This pattern suggests that tree frogs did not use land-bridges across the Korea Strait, which connected southern Japan with the Korean mainland during the last glacial cycles (Fig. 5; [16]), and thus remained isolated throughout the Quaternary. Alternatively, oversea dispersal may have occurred but without significant genetic consequences for the tree frog populations persisting on both sides of the Korea Strait. Indeed, the environmental conditions seem to have remained suitable on the southern Korean Peninsula and in Japan during the LGM, probably enabling long-term persistence of large populations (Fig. 5). For example, it seems possible that tree frogs may have formed admixed populations on former Japan Sea land-bridges, which subsequently disappeared, when water levels rose up during interglacials. It remains unclear whether the presence of a continental nuclear haplotype on Kyushu Island (pop. 10) results from recent oversea(s) dispersal or ancestral polymorphism. Accordingly, other terrestrial vertebrates show little evidence of recent colonization from the continent towards the Japanese Archipelago [16].

Contrasting with our phylogenetic data, previous allozyme genetic distances [52] suggested stronger differentiation between Korean and Japanese populations than between southern and northern Japanese populations, which was interpreted as an earlier split from continental *Hyla* [52]. However, the Korean sample in this study might actually represent *H. immaculata*/*H. suweonensis* (collected from the type locality of *H. suwoenensis*, Suweon). Additional nuclear loci will help solving the nuclear phylogeny of *H. japonica* clades.

Interestingly, the phylogeographic pattern of *H. japonica* can also be considered as a case of ring diversification. This type of diversification involves parapatric or allopatric differentiation of populations along a stepping-stone-like geographic continuum around a major dispersal barrier, preventing gene flow between non-neighbouring demes. In our case, populations diversified around the Sea of Japan, stretching from Sakhalin to southern Honshu on the one hand (n. t. A), and from Eastern Russia, China, Korea and onto the Southern Japanese Archipelageo on the other hand (n. t. B). As recently emphasized, ring-like phylogeographic patterns might be more common than previously assumed [55, 56], especially in regions with complex topography and Pleistocene climatic instability [57]. Ancestral ring diversifications can sometimes also be later “broken into multiple species presumed to be evolving independently, usually obscuring the evolutionary dynamics that generate diversity” [58, 59], a scenario that would be in line with the split and seemingly independent history of our two separate taxa n. t. A and B around the Sea of Japan. Future population genetic studies might help to determine the role of isolation-by-distance in shaping *H. japonica*’s diversity around the Sea of Japan and test whether the northern, terminal mtDNA lineages A1 (n. t. A) and B5 (n. t. B) interact and “close” this ring (i.e. between our loc. 1 and 28). Moreover, this pattern should be tested in other organisms, where cryptic diversity may have arisen by similar biogeographic processes.

### Late Quaternary processes on the continental east-Asia

The distribution of the two continental *H. japonica* lineages (subclades B4 and B5) supports multiple glacial refugia across the Korean Peninsula. In this region, glacial conditions were supposedly milder (Fig. 5), but topography (the Taeback Mountains) and insularity (Tsushima and Jeju Islands, pop. 11–12) likely promoted population structure, as previously shown in South-Korean tree frog populations [60]. Based on haplotype distribution within B5, where different mitochondrial haplogroups segregate in Korea (B5a, B5b), and in northern ranges (B5c, Fig. 3), suggesting an additional refugium further north, we provide additional evidence for recent continent-scale population differentiation. This hypothetical refugium was probably located along the North-Korean and Russian coast (as suggested by the CCSM past distribution model, Fig. 5), from which Transiberian China, Russia and Mongolia were recolonized postglacially. Accordingly, the B5c mtDNA-haplogroup features signs of recent expansion (star-like pattern in haplotype networks); in addition, northern populations (pop. 24–34) are genetically homogeneous, which likely testifies of the loss of refugial diversity due to expansion-associated drift [61]. Generally, these results support that glacial cycles, supposedly milder in NE-Asia compared to other parts of the Palearctic, significantly influenced the distribution of genetic diversity in this region, in line with recent studies on other animals [8] and plants [13, 14].

The lack of sequence differentiation between the Chinese *H. immaculata* and Korean *H. suweonensis*, which share identical nuclear haplotypes, suggests a very recent divergence despite current allopatric distributions (Fig. 6). Thus, populations seem to have been connected recently, in the Late Quaternary, probably through Late Pleistocene land-bridges across the shallow Yellow Sea. Indeed, marine geological data suggested that during the last glacial period, the Yellow Sea’s level was about 120 m lower than at present day [62], and still more than 50 m lower at the beginning of the Holocene [63], which would enable such connections and exchanges [64, 65], particularly off the Shandong Peninsula, separated from Korea mainland by only 250 km. This probably large land-bridge is illustrated from our LGM distribution models for *H. japonica* (Fig. 5). Future additional sampling across the Chinese mainland range parts may help testing whether this Yellow Sea land-bridge still acted as a dispersal filter: in our limited dataset, the two *H. immaculata* mtDNA-haplotypes (IMM01, IMM02) were not shared by any Korean *H. suweonensis*, suggesting limited dispersal and/or recent divergences.

### Taxonomic implications and nomenclatural issues

Our results stress the need for systematic revisions of East-Asian tree frogs. First, we provide evidence for cryptic speciation within *H. japonica* (Günther, 1859), which can be considered a superspecies, including at least two main taxa (our clades A and B, corresponding to two new taxa, n. t. A and n. t. B). Our findings are consistent with Li et al*.* [5], who isolated a distinct mitochondrial lineage from few specimens on Sakhalin and Kunashir Islands (our mtDNA-clade A, subclade A1, corresponding to “*H. japonica* A” in [5]), early-diverged from other populations (our mtDNA-clade B, “*H. japonica* B” in [5]). The old split between these two mtDNA-clades (~5 Mya), combined with their concordant nuclear differentiation, should warrant them specific status. Other Palearctic *Hyla* lineages of similar or lower degree of divergence are recognized as different species [28, 66], and hardly hybridize in their secondary contact zones [67].

Second, there has been an ongoing debate on whether continental *H. japonica* populations should be considered a separate species, *H. stepheni* (or its junior synonym *H. ussuriensis*), distinct from Japanese ones [27]. Based on slower evolving markers (12S and 16S rDNA), Li et al*.* [5] argued that *H. japonica* and *H. ussuriensis* can be considered synonyms. In contrast, our present data rather suggest that these continental and southern Japanese tree frogs form monophyletic mitochondrial lineages, as old as about 3 Mya (our mtDNA-subclades B1, B2-B3 and B4-B5), with distinct, although paraphyletic, nuclear haplogroups. Interestingly, experimental crosses between “*H. ussuriensis*” (from mainland Korea and Tsushima island) with southern Japanese “*H. japonica*” (from Hiroshima) suffered from lower metamorphosis rates and inferior offspring fitness compared to controls, which might indicate post-zygotic isolation [68]. Additional multilocus genetic surveys, as well as morphological and bioacoustic characterization, will inform about further reaching taxonomic and nomenclatural decisions. At present, these continental and island populations should be considered independent genetic entities for wildlife management. The same is true for several northern Japanese lineages within mtDNA-clade A that show allopatric distributions and features similar amounts of mitochondrial divergence (e.g. A1, A2-A3, A4).

The split of the nominal *H. japonica* into two taxonomic entities (n. t. A and n. t. B) raises the question to which one the species-epitheton *japonica*, coined by Günther ([24], “1858”), belongs to. Despite intense search we were unable to narrow down the type region “Japan” to a specific locality at which the type series was collected (syntypes, BMNH 44.2.22.107; 3 specimens). According to Günther (p. 109 in [24]) as well as according to labels on the collection jars and in the hand-written BMNH catalogue (Streicher, pers. comm., I/2016) these specimens were obtained from the Leiden (“Leyden”) collection, and thus most probably were collected by Siebold and Bürger [69]. Indeed, the Leiden catalogue mentions the arrival of tree frogs (as “*Hyla arborea*”) with database record “RMNH.RENA.1701, *Hyla arborea*, 17 ex., collected by: Siebold & Bürger, locality: Japan” (Dondorp, pers. comm., XII/2015). While these frogs are extensively described by Temminck and Schlegel (p. 112 in [69]) and depicted (p. 312 in [69]; as “*Hyla arborea*”), their work contains no further geographic details. Siebold was a resident physician at the Dutch trading post on Dejima Island in Nagasaki. Yet, in 1826, he traveled at least to Edo (now Tokyo). Therefore, types of *H. japonica* may have originated from the vicinities of Nagasaki (inhabited by n. t. A), but it cannot be excluded that the types were collected in the vicinities of Tokyo (inhabited by n. t. B), or elsewhere in Japan, as zoological and botanical material was also obtained from local people from several parts of Japan [69, 70]. In conclusion, it is therefore possible that either of the two taxa (n. t. A or n. t. B, or even both) could be represented in the type series. Without further details through historical research in the London and Leiden collections, such as additional travel documents left by Siebold or genetic research involving the type series, the nomenclatural question to which lineage the name *japonica* belongs, cannot be conclusively answered.

Finally, both our mitochondrial and nuclear datasets unambiguously supported that *H. immaculata* and *H. suweonensis* belong to one and the same tree frog species, as recently suspected based on few mitochondrial sequences [5]. From our data, these names can be declared synonymous, leaving *H. suweonensis* Kuramoto, 1980 [26] a junior subjective synonym of *H. immaculata* Boettger, 1888 [25]. This lineage forms a sister taxon to the *H. japonica* superspecies. The use of faster-evolving markers, such as microsatellites, will allow to better understand the recent history of *H. immaculata* populations, and define management units for their conservation.

## Conclusions

The phylogeography of Eastern tree frogs revealed cryptic diversity in a ring-like manner, and potential speciation of several deeply-diverged lineages. These divergences likely result from past tectonic events and paleoclimatic changes, and their effects on the formation and disappearance of land-bridges between the topographically complex Japanese Archipelago and the Korean Peninsula, paralleling the situation in other terrestrial vertebrates. Furthermore, by the identification of multiple glacial refugia and continental routes of post-glacial recolonization, we demonstrated that Quaternary glaciations also had a strong genetic impact on the evolution of East-Asian anuran amphibians. For this part of the Palearctic realm, it is usually assumed that such effects are limited compared to other biogeographic regions of the northern hemisphere.

## Additional files

Additional file 1: Origin of the samples included in this study (XLSX 18 kb)
Additional file 2: Maximum credibility tree from the mitochondrial dating in BEAST. (PDF 201 kb)
Additional file 3: List of *H. japonica* occurrences used in the distribution models (XLSX 35 kb)
Additional file 4: Mitochondrial genetic distances (*Dxy*) between and within Eastern tree frog taxa. (PDF 32 kb)

## Abbreviations

My: Million years
Mya: Million years ago
