# Supplementary material for: Phylogeography reveals an ancient cryptic radiation in East-Asian tree frogs (Hyla japonica group) and complex relationships between continental and island lineages
Source: BMC Evol Biol. 2016 Nov 23;16:253. doi: 10.1186/s12862-016-0814-x (PMC5121986; doi:10.1186/s12862-016-0814-x)

Additional file 2: Maximum credibility cladogram from the mitochondrial dating analysis in BEAST.
Calibration points in the European radiation are indicated by arrows, following estimates from Stöck *et al.* [25].

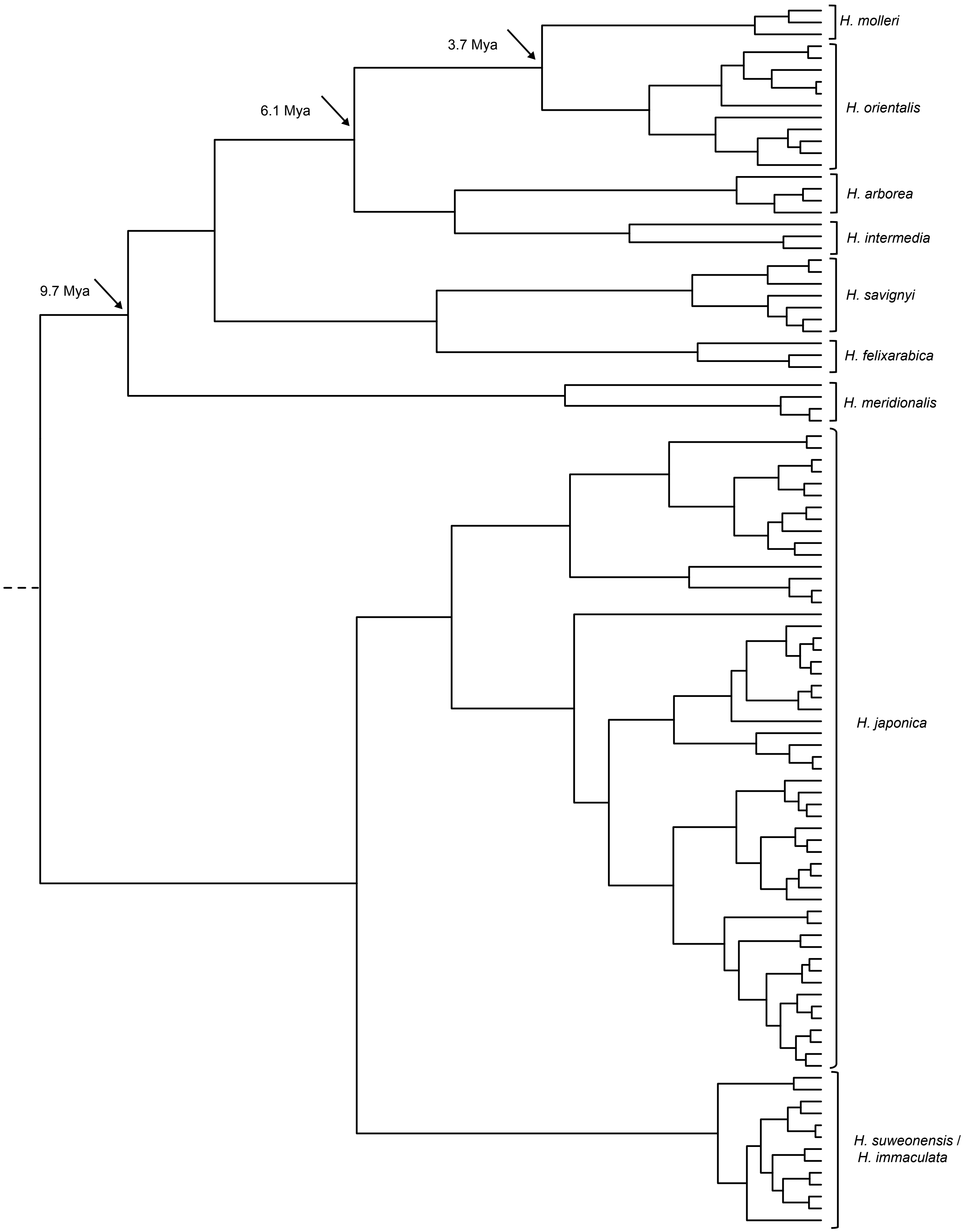

Supplement: Additional file 2: — Maximum credibility tree from the mitochondrial dating in BEAST. (PDF 201 kb) [file 12862_2016_814_MOESM2_ESM.pdf]
