# Supplementary material for: Phylogeography reveals an ancient cryptic radiation in East-Asian tree frogs (Hyla japonica group) and complex relationships between continental and island lineages
Source: BMC Evol Biol. 2016 Nov 23;16:253. doi: 10.1186/s12862-016-0814-x (PMC5121986; doi:10.1186/s12862-016-0814-x)
Supplement: Additional file 4: — Mitochondrial genetic distances (Dxy) between and within Eastern tree frog taxa. (PDF 32 kb) [file 12862_2016_814_MOESM4_ESM.pdf]

Additional file 4: Mitochondrial genetic distances (*Dxy*) between and within Eastern tree frog taxa

Between taxa

	H. immaculata /*H. suweonensis*	*H. japonica* A	*H. japonica* B
H. immaculata / *H. suweonensis*	-		
H. japonica A	0.135	-	
H. japonica B	0.128	0.104	-

Within *H. japonica* taxon A

	A1	A2	A3	A4
A1	-			
A2	0.036	-		
A3	0.066	0.055	-	
A4	0.070	0.064	0.036	-

Within *H. japonica* taxon B

	B1	B2	B3	B4	B5
B1	-				
B2	0.036	-			
B3	0.039	0.021	-		
B4	0.041	0.035	0.034	-	
B5	0.042	0.036	0.036	0.017	-
